# EMR in Eye Units

**Published:** 2020

**Authors:** Georgiana Camburu, Victor Lorin Purcărea

**Affiliations:** * “Carol Davila” University of Medicine and Pharmacy, Bucharest, Romania

**Keywords:** electronic medical record, electronic medical record in Eye Unit

## Abstract

There are some questions that have to be asked when considering if EMR is suitable for a National Health System: First the Viability, Reliability, and Integrity of the EMR. When thinking about EMR, it has to be compounded based on some standards, that can be taken from other Health Systems or own. Secondly, the proper type of Ophthalmic EMC System (Web based EMR/ Client Server EMR) should be taken into consideration.

Another question that raises is if EMR can not only be specialized in Ophthalmology, but also Subspecialties such as Glaucoma, Retina and other. This offers a possibility of completing a Database that can be used for Studies and Audit and improve the Health System. Using EMR can also offer accurate data that can estimate the prevalence of some specific conditions and rare diseases.

Electronic Medical Record (EMR) is a systematized collection of patient health information that is electronically stored in digital formats [**1**].

Another definition was offered by the Institute of Medicine (US) Committee on Improving the Patient Record, but instead they used the term Computer-based patient record as an electronic patient record that resides in a system specifically designed to support users by providing access to complete and accurate data, alerts, reminders, clinical decision support systems, links to medical knowledge, and other aids [**3**]. Here, the term tries to offer a full picture of the whole process: collecting, entering and analyzing the data.

The history of EMC started in the mid-1960s, when Lockheed developed an electronic system known then as the first clinical information system [**2**]. Previous to this, patients kept all their health information (lab reports, visit notes, medications) on sheets of paper that were labeled using the patients’ names. 

There are some questions that have to be asked when considering if EMR is suitable for a National Health System: first the Viability, Reliability, and Integrity of the EMR. When thinking about EMR, it has to be compounded based on some standards that can be taken from other Health Systems or own. Secondly, the proper type of Ophthalmic EMC System (Web based EMR/ Client Server EMR) should be taken into consideration.

Another question that raises is if EMR can not only be specialized in Ophthalmology, but also in Subspecialties such as Glaucoma, Retina and other. This offers a possibility of completing a database that can be used for studies and audit and improve the Health System. Using EMR can also offer accurate data that can estimate the prevalence of some specific conditions and rare diseases. 

When talking about Viability, Reliability and Integrity of the EMR, first it has to be decided if it is part of a hospital informational system, with both functions: clinical and administrative or medical information system, just collecting, storing, and making available the data important for the patient care. The aim of this type of system is not that of facilitating financial information, but instead focusing on improving patient care [**3**]. As a system, the viability of the EMR depends on the Operational effectiveness - having those resources needed for accurate patients’ records, Financial effectiveness - trying to maximize the income and to reduce the loss of resources, Compliance - ensuring the function of the system based on laws and regulations.

The quality of the system is often missed or ignored in medical practices. The quality of EMR can be measured by the Integrity and Reliability of the system. A reliable EMR should provide the right information to the right user at the right moment. The EMR should offer both the physician and the patient the information desired.

Reliability is related to Integrity. Some records can be illicitly modified to maximize the income for a procedure that cost less or never happened. Integrity of the system means that the data is stored without missing records, multiple records of the same event, records that contain incorrect information or events that never happened [**4**]. 

## Integrity is based on Standards

The Certification Commission for Healthcare Information Technology is a federal nonprofit government contractor that certifies EMR systems. The Certification is offered after evaluating the functionality, security and interoperability, or the capacity of the systems to communicate with one another and incorporate digital imaging in the patient record.

The Eye Care Working Group, funded by AAO, is a group of clinicians, users, vendors, researchers, that stipulates the Digital Imaging and Communications in Medicine (DICOM) Standards for Eye practice [**5**]. 

Next, the type of EMR System we want to use has to be chosen. By choosing Application Service Provider Web based EMR, the data is stored online, so the information cannot be freely accessed at any moment in time, being controlled by the provider. Moreover, the provider can update the soft or use the data stored without consent. Other limitations are the necessity of an internet connection, slow performance during the peak times during the day, or slow downloads. Client Server EMR system has high performances. The user does not need an Internet Connection, so it has high speed. The disadvantage is the low interoperability between systems with different software losing precious data. Recently, another type of system appeared, fulfilling the previous systems limitations. By using locally hosted or Hybrid option EMR, the user can have both the software and access the data whenever needed. Nevertheless, the user has to purchase his own server and connect to it via Internet, when outside of the office [**6**].

Further, EMRs have not only been designed particularly for Eye care, but also for subspecialties. This type of EMR can be suitable for a Department or Sub-specialized Clinic. Using EMR in Pediatric Ophthalmology is a challenge because it combines the particularities found in Ophthalmology, heavy use of imaging and documentation, and also Paediatric particularities as changes that occur in developmental stages. In a survey undergone to reveal the benefit of routine EMR used by paediatric ophthalmic clinicians, participants had to choose from a list of 18 benefits. The mean was 8.59/ per participant. The limitation previously mentioned can explain the low usage of EMR in the ophthalmic paediatric field [**7**]. Novack analyzed the data gathered from the patients who have suffered retinal detachment provided by EMR. The conclusion was that only 41% of the hospitals in the US have electronically available medical information from outside providers or sources when treating a patient, physicians in outpatient settings far worse, only 14% of the office-based physician sharing data with providers outside their organization [**8**]. Reisman offers an explanation to this result, referring to the lack of standards among different ERH vendors. Clinicians can store and analyze previous measurements, for example Central Corneal Thickness, when needed.

One of the pros. of using EMRs is that they provide a rich database, from where the user is able to reach some conclusion and improve the medical care. A study undergone by N. Restrepo showed that the user is able to extract Primary Open Angle Glaucoma from EMR for genetic associations. In other words, he created an algorithm that can extract a specific clinical subtype of glaucoma from EMRs in the absence of digital photographs [**9**]. Gignac illustrated the Contribution of EMR to the management of rare diseases. His conclusion was that the use of specific EMR might improve surveillance of rare diseases and sharing of data between centers will support clinical research and innovation [**10**].

In order to choose the applicability of EMR, a metanalysis can be performed on some studies undergone in the health systems from different countries. In a study made in the US about long term financial and clinical impact of EHR on Academic Ophthalmology Practice it appeared that the difference in inflation did not change significantly before and 9 years after the EHR implementation [**11**]. Another study about the use of EHR and administrative data for public health surveillance of the eye health and vision related conditions in the US showed that EHR can offer additional information to make “a comprehensive vision” and eye health surveillance with both disadvantages and advantages [**12**]. The cross-sectional study about the distribution and extent of EMR in eye units across UK suggested that it is pleasing to see numerous eye units that are using EMR (45.3% currently using, 26.4% implementing), but the concept still needs further optimization for the records system to allow proper data transfer between units.

To eliminate some of these differences, the Royal Collage of Ophthalmologists defined a minimum of standards indicated for the vendors as “a must”.

Another aspect that can be reflected on is the satisfaction of the user/ system members in managing EHR, which in our case, is the physician or the patient. A descriptive qualitative study that entailed the physician’s satisfaction with EMR in health centers from Al Ain United Arab highlighted that the physicians are satisfied with EMR and have a good perception about it [**13**]. In a pilot study regarding the amount of time the ophthalmologists using EHR spend examining the patient and correlated with the patient’s satisfaction, demonstrated that even if the ophthalmologist spent one-third of time looking at the computer, the patient satisfaction levels were very high.

## Conclusion

Our opinion is that the implementation of the EMR is the National Health System is a fundamental step in the progression and optimization of the Eye Care.
